# Inhibition of G-Protein βγ Signaling Enhances T Cell Receptor-Stimulated Interleukin 2 Transcription in CD4^+^ T Helper Cells

**DOI:** 10.1371/journal.pone.0116575

**Published:** 2015-01-28

**Authors:** Evan A. Yost, Thomas R. Hynes, Cassandra M. Hartle, Braden J. Ott, Catherine H. Berlot

**Affiliations:** Weis Center for Research, Geisinger Clinic, Danville, Pennsylvania, 17822-2623, United States of America; Hanyang University, REPUBLIC OF KOREA

## Abstract

G-protein-coupled receptor (GPCR) signaling modulates the expression of cytokines that are drug targets for immune disorders. However, although GPCRs are common targets for other diseases, there are few GPCR-based pharmaceuticals for inflammation. The purpose of this study was to determine whether targeting G-protein βγ (Gβγ) complexes could provide a useful new approach for modulating interleukin 2 (IL-2) levels in CD4^+^ T helper cells. Gallein, a small molecule inhibitor of Gβγ, increased levels of T cell receptor (TCR)-stimulated IL-2 mRNA in primary human naïve and memory CD4^+^ T helper cells and in Jurkat human CD4^+^ leukemia T cells. Gβ_1_ and Gβ_2_ mRNA accounted for >99% of Gβ mRNA, and small interfering RNA (siRNA)-mediated silencing of Gβ_1_ but not Gβ_2_ enhanced TCR-stimulated IL-2 mRNA increases. Blocking Gβγ enhanced TCR-stimulated increases in IL-2 transcription without affecting IL-2 mRNA stability. Blocking Gβγ also enhanced TCR-stimulated increases in nuclear localization of nuclear factor of activated T cells 1 (NFAT1), NFAT transcriptional activity, and levels of intracellular Ca^2+^. Potentiation of IL-2 transcription required continuous Gβγ inhibition during at least two days of TCR stimulation, suggesting that induction or repression of additional signaling proteins during T cell activation and differentiation might be involved. The potentiation of TCR-stimulated IL-2 transcription that results from blocking Gβγ in CD4^+^ T helper cells could have applications for autoimmune diseases.

## Introduction

G protein-coupled receptor (GPCR) signaling exerts multiple influences on cytokine levels with vast implications for immunodeficiency and autoimmune diseases [1]. However, although GPCRs are fairly common drug targets for neurological and cardiovascular diseases, there are fewer examples in the field of immune disorders. Of the 73 GPCRs thought to have a function in inflammation, only two so far have been successful drug targets for inflammatory disorders, yielding therapeutics for asthma (CysLT-1 receptor) and allergic rhinitis (H1 histamine receptor) [2]. Although chemokine receptors, which regulate the migration of immune cells, have been a major focus for drug development, only two, a CCR5 inhibitor and a CXCR4 antagonist, are registered drugs, but not for autoimmune diseases [3]. As there are multiple ligands for individual chemokine receptors and multiple receptors for particular chemokines, targeting chemokine signaling downstream from the chemokine receptors may potentially have greater therapeutic efficacy than blocking a single one [4]. Similarly, while targeting GPCR signaling to regulate cytokine levels may well prove to be a useful therapeutic approach, targeting signaling distal to the GPCRs may also be advantageous, as multiple GPCRs can influence cytokine levels.

IL-2 is a growth factor for both effector and regulatory T cells and can have both positive and negative effects on immune responses [5]. Although IL-2 has been used to augment immune responses to treat cancer [6] and persistent viral infections [7], it also effectively suppressed immune responses in chronic graft-versus-host disease [8] and hepatitis C virus-induced vasculitis [9]. One potential explanation for these apparently discrepant effects is that the dose of IL-2 determines the effect, with low doses preferentially stimulating regulatory T cells and high doses preferentially amplifying effector T cells [5]. The current strategy of low-dose IL-2 therapy for autoimmune diseases consists of daily subcutaneous administration of recombinant IL-2 [8,9]. The effectiveness of this approach may be limited by the very short half-life of exogenous IL-2 *in vivo*, reported to be ∼7 min [10]. As effector T cells are the primary source of IL-2 [11], increasing their production of IL-2 might provide a more nuanced approach to treatment in terms of the localization and duration of IL-2 production compared with injection of IL-2.

Several GPCRs have been shown to decrease TCR-stimulated IL-2 levels via distinct pathways that may involve both G-protein α and βγ subunits. TCR-stimulated increases in IL-2 in CD4^+^ T lymphocytes were inhibited by stimulation of the G_s_-coupled A_2A_-adenosine [12] and β_2_-adrenergic receptors [13]. Increasing cAMP had the same effect as activation of the A_2A_-adenosine receptor [12]. In contrast, activation of the β_2_-adrenergic receptor reduced TCR-stimulated IL-2 production via inhibition of calcineurin activity. Receptor-independent increases in cAMP were not sufficient to inhibit calcineurin, which required interaction between PKA and A-kinase anchoring protein [13]. Increased cAMP/PKA activity also mediated inhibition of TCR-stimulated IL-2 upon activation of the G_i/o_-coupled μ opioid [14] and CB_1_ and CB_2_ cannabinoid receptors [15] in T cells. As Gβγ from G_i/o_ can stimulate certain isoforms of adenylyl cyclase [16], Gβγ could be involved in these responses. Additionally in CD4^+^ T lymphocytes, the Edg-4/LPA_2_ receptor inhibited, while the closely related Edg-2/LPA_1_ receptor potentiated TCR-stimulated IL-2 [17]. Both of these receptors couple to G_i/o_, G_q/11_, and G_12/13_, and signal to multiple downstream molecules via pathways that could involve both Gα and Gβγ [18].

The purpose of this study was to determine whether directly targeting Gβγ could provide a useful new approach to modulate IL-2 levels in CD4^+^ T helper cells. Gβγ complexes mediate numerous signaling pathways downstream of GPCRs [19] and have proven to be feasible drug targets [20]. For instance, a class of Gβγ inhibitors, which includes the structurally related compounds gallein and M119, specifically blocks interactions between Gβγ, but not Gα, with effectors, and does not promote dissociation of Gα from Gβγ [21]. Although relatively little is known about the role of Gβγ complexes in modulating T cell signaling, gallein/M119 has been used successfully in animal models to inhibit neutrophil chemotaxis and inflammation [22]. Gallein/M119 has also been used successfully in animal models to potentiate morphine-induced analgesia [21] and to inhibit the progression of heart failure [23]. These precedents suggested that targeting Gβγ might provide an effective way to block signaling from the multiple GPCRs that can inhibit TCR-stimulated IL-2 increases. Indeed, this study demonstrates that inhibiting Gβγ enhances TCR-stimulated IL-2 transcription.

## Materials and Methods

### Plasmids

For luciferase reporter assays, the human IL-2 promoter from −300 to 0 bp was synthesized by Life Technologies and subcloned as a HindIII cassette into pGL3 (Promega). AP1(1), NF-AT, and NFκB(2) luciferase reporter vectors, which contained transcription factor binding motifs that monitored activation of the respective transcription factors (Affymetrix/Panomics), were also subcloned into pGL3. pRL-TK Renilla (Promega) was used to normalize luciferase activities.

For live cell imaging of NFAT1 and NFAT2, GFP fusion constructs were used. For NFAT1, HA-NFAT1(1-460)-GFP (Addgene plasmid 11107) from the laboratory of Anjana Rao [24], consisting of the amino terminal regulatory domain, but lacking an intact DNA-binding domain of NFAT1, fused to the amino terminus of GFP was used (referred to as NFAT1-GFP). For NFAT2, EGFPC1-huNRATc1EE-WT (Addgene plasmid 24219) from the laboratory of Jerry Crabtree [25], consisting of the amino terminal regulatory domain, but lacking an intact DNA-binding domain of NFAT2, fused to the carboxyl terminus of GFP was used (referred to as GFP-NFAT2). Monomeric Cherry (mCherry) [26] was obtained from Roger Tsien (University of California, San Diego, CA).

For live cell imaging of intracellular Ca^2+^, a fusion of the red intensiometric Ca^2+^ indicator (R-GECO1) from the plasmid, CMV-R-GECO1 (Addgene plasmid 32444) from the laboratory of Robert Campbell [27], to monomeric Cerulean (mCerulean) [28] was used. mCerulean (obtained from David Piston, Vanderbilt University, Nashville, TN) was amplified by a PCR reaction that added BamHI sites to each end and then subcloned into BamHI-digested CMV-R-GECO1, resulting in a fusion of mCerulean to the amino terminal end of R-GECO1, referred to as mCerulean-R-GECO1.

### Ethics statement and study population

This study was reviewed and approved by the Geisinger Health System Internal Review Board, and all study participants signed informed consent. Peripheral blood was obtained from 30 healthy women 18 to 70 years old who did not have any autoimmune, infectious, or atopic diseases, clinical suspicion of anemia, or treatment with greater than 10 mg of prednisone within 12 hours of the blood draw.

### Isolation and culture of human CD4^+^ T cells and Jurkat T cells

Peripheral blood mononuclear cells were isolated using Ficoll-Paque density gradient centrifugation. CD4^+^ T cells were isolated by depletion of non-CD4^+^ T cells using a CD4^+^ T Cell Isolation Kit II (Miltenyi Biotec). The cells were then separated into naïve and memory CD4^+^ T cells using a Naïve CD4^+^ T cell Isolation Kit (Miltenyi Biotec). Purification of the cells was confirmed by labeling samples before and after purification with fluorescently labeled antibodies to either CD4 and CD45RA (to label naïve cells) or CD4 and CD45RO (to label memory cells) and analysis using flow cytometry. 94.3% of the cells in the naïve T cell preparations were CD4^+^ (SE = 0.7%, ranging from 83.9% to 98.6%) and 83.8% were CD45RA^+^ (SE = 1.4%, ranging from 68.1 to 95.9%). 95.2% of the cells in the memory T cell preparations were CD4^+^ (SE = 0.4%, ranging from 89.7% to 98%) and 75.0% were CD45RO^+^ (SE = 1.8%, ranging from 55.0 to 88.6%). Cells were plated in 24-well dishes coated with 2.5 μg/mL anti-CD3 antibody (Miltenyi) in RPMI containing 10% FCS, 2.5 μg/mL anti-CD28 antibody (Miltenyi) and IL-2 (2 ng/mL) (R&D Systems). For Type 1 T helper cell (TH1) differentiation, the media also included 20 ng/mL IL-12 and 1 μg/mL anti-IL-4 antibody (R&D Systems). For Type 2 T helper cell (TH2) differentiation, the media also included 20 ng/mL IL-4 and 2 μg/mL anti-IL-12 antibody (R&D Systems). Cells were harvested after three days.

Jurkat T cells (Clone E6-1) were obtained from ATCC and cultured in RPMI containing 10% FCS. For TCR activation, the cells were grown in wells coated with anti-CD3 (2.5 μg/mL) in the presence of soluble anti-CD28 (2.5 μg/mL).

### siRNA and gallein treatments

siRNAs were produced by Dharmacon. The sequence of Gβ_1_ siRNA is from [29] and is directed at bp 351–379: GGAUAACAUUUGCUCCAUU. A second Gβ_1_ siRNA, si β_1_(8), directed at a different region of the Gβ_1_ sequence, was designed by Dharmacon (ON-TARGETplus SMARTpool siRNA J-017242-08, GNB1). The sequence of the β_2_ siRNA was: GUGGAGAUAAGAAGGGGAUUU. The non-targeting (NT) siRNA used was ON-TARGETplus Non-targeting Pool (Dharmacon, D-001810-10-20).

siRNAs were introduced into primary CD4^+^ T cells and Jurkat T cells by nucleofection using a Nucleofector II Device (amaxa/Lonza). 2–9 × 10^6^ primary CD4^+^ T cells were nucleofected with 10 μM siRNA using 100 μL of Human T Cell Nucleofector Solution and Program U-014. After nucleofection, the primary CD4^+^ T cells were incubated in RPMI with 10% FCS for 6 hours before transfer to activating/differentiating media. 4 × 10^6^ Jurkat cells were nucleofected with 10 μM siRNA in 100 μL of Cell Line Nucleofector Solution V using Program X-005. Nucleofected Jurkat cells were transferred immediately into activating media.

Gallein and fluorescein (TCI America) were used at a final concentration of 15 μM.

### Quantitative PCR (qPCR)

RNA was prepared using RNeasy Plus Mini Kits (Qiagen). cDNA was prepared using QuantiTect Reverse Transcription kits (Qiagen). QPCR was performed using TaqMan Gene Expression Assays (Applied Biosystems) and an Applied Biosystems qPCR machine. mRNA expression levels were determined by comparing the C_t_ value of the mRNA of interest to that of the house-keeping gene GAPDH in the same preparation.

### IL-2 ELISA

IL-2 secreted into the media by Jurkat cells was quantified using a human IL-2 Quantikine ELISA kit (R&D Systems).

### Immunoblots

For Gβ_1_ and Gβ_2_ immunoblots, Gβ_1_ (XAB-00301-1-G) and Gβ_2_ (XAB-00401-1-G) antibodies from CytoSignal, LLC were used to detect expression in membranes prepared as described [30]. For determination of NFAT1 and NFAT2 expression, total cell lysates were used. NFAT1 antibody (ab2722) and NFAT2 antibody (ab2796) were obtained from Abcam Inc.

7 μg of membrane proteins or total lysates were resolved on NuPAGE 4–12% Bis-Tris gels and transferred to Invitrolon PVDF membranes (Life Technologies). The antigen-antibody complexes were detected using SuperSignal West Pico or Fempto Chemiluminescent Substrate (Pierce Biotechnology, Inc.). Chemiluminescence was imaged using a Fuji LAS-4000 Luminescent Image Analyzer. Bands in the images were quantified using ImageJ software.

### Actinomycin D assay

Jurkat cells were nucleofected with Gβ_1_ siRNA or NT siRNA and stimulated with plate-bound anti-CD3 and soluble anti-CD28 for three days as described above and then treated with 10 μg/mL of Actinomycin D to inhibit transcription. After incubation with Actinomycin D for 0, 5, 10, 20, or 30 minutes, the cells were removed from the wells and RNA was prepared.

### Luciferase Assay

Jurkat cells were nucleofected with 2 μg of a luciferase reporter plasmid and 0.1 μg of pRL-TK Renilla and then stimulated or not with plate-bound anti-CD3 (2.5 μg/mL) and soluble anti-CD28 (2.5 μg/mL) in the presence or absence of 15 μM gallein. The Dual-Luciferase Reporter Assay System (Promega) was used according to the manufacturer’s instructions and data were collected using a POLARstar Optima plate reader.

### Imaging and analysis of NFAT-GFP fusions in live Jurkat cells

4 × 10^6^ Jurkat cells were suspended in 0.25 mL of HEPES buffered RPMI media with no serum. Plasmids expressing NFAT1-GFP (10 μg) or GFP-NFAT2 (10 μg) and mCherry (2 μg) were introduced into the cells by electroporation using 250 V and 330 μF capacitance with a BRL Cell-Porator. Cells were immediately transferred to 4 mL of bicarbonate buffered RPMI media containing 5% FCS at 37°C. After 30 min, cells were plated at a density of 3.5 × 10^5^ cells per well in 250 μL on Lab-Tek II, 8 well chambered coverslips. For TCR-activating conditions, wells were pre-coated with 2.5 μg/mL anti-CD3 and soluble anti-CD28 was added to a final concentration of 2.5 μg/mL.

Cells were imaged three days after electroporation using a white light spinning disc confocal microscope comprised of an Olympus IX81 inverted microscope, UIS2 60× 1.42 N.A. objective, IX2-DSU spinning disc system, 100 watt mercury arc lamp, Hamamatsu C9100-02 electron multiplier camera, Ludl filter wheels, shutters, and xy stage, under the control of IPLab software (BD Biosciences). Excitation and emission filters for NucBlue (430/24, 470/24), GFP (470/40, 520/40), mCherry (572/35, 632/60), were obtained from Chroma. A stage incubator was used to maintain the cells at 37°C with 5% CO_2_. One hour prior to imaging, 10 μL of NucBlue Live ReadyProbes Reagent (Life Technologies) was added to each well to label the nucleus. Unstimulated cells were transferred to wells that had been coated with 0.1 mg/mL poly-L-lysine for 30 min, and then imaged after 30 min. The criteria for selecting cells for imaging were visible expression of all transfected fluorescent constructs, and for TCR-stimulated cells, clear spreading on the coverslip, indicative of activation. For each condition, cells from at least five independent transfections were imaged.

All image processing was performed using ImageJ software. The background intensity, determined by averaging the intensity of a region of pixels outside the cell, was subtracted from each image. The mCherry image was thresholded to determine the pixels corresponding to the whole cell. The NucBlue image was thresholded to determine the pixels corresponding to the nucleus. The cytoplasmic pixels were determined by subtracting the nuclear pixels from the whole cell pixels. The average intensities of the nuclear and cytoplasmic pixels in the GFP image of NFAT1 or NFAT2 were determined and the nuclear to cytoplasmic intensity ratio was calculated.

### Imaging and analysis of intracellular Ca^2+^ in live Jurkat cells using mCerulean-R-GECO1

The mCerulean-R-GECO1 plasmid (20 ug) was introduced into Jurkat cells by electroporation and the cells were plated and stimulated with plate-bound anti-CD3 and soluble anti-CD28 as described above for the NFAT-GFP imaging experiments. Stimulated cells were imaged one or three days after electroporation using the Olympus IX81 inverted microscope. Excitation and emission filters for CFP (430/24, 470/24) and mCherry (572/35, 632/60) were obtained from Chroma. For each field imaged, a cell displaying clear spreading on the coverslip was found. As individual cells displayed different amounts of spreading, one plane of focus was not optimal for analysis of all cells in a field. Therefore, starting at the bottom of the cover slip, ten 0.75 uM z-sections were taken of each field. Cells were analyzed if they had intensity in the first z-section, indicating that they were in contact with the coverslip. Unstimulated cells were imaged three days after electroporation and were transferred to untreated wells before imaging. Since they did not spread on the untreated coverslip, a single exposure was taken for each cell and all cells were analyzed. For each condition, cells from at least three independent transfections were imaged.

All image processing was performed using ImageJ software. The background intensity, determined by averaging the intensity of a region of pixels outside the cell, was subtracted from each image. The mCerulean image was thresholded for each z-section to determine the pixels corresponding to the whole cell. For each cell, the z-section that had the largest amount of thresholded mCerulean pixels was used for analysis. The average intensities of the whole cell pixels in the mCerulean and R-GECO1 images were determined and the R-GECO1/mCerulean ratio was calculated.

### Statistics

The significance of effects of siRNAs, gallein, and fluorescein on primary CD4^+^ T cells was determined using the Wilcoxon signed rank test (paired, non-parametric). The significance of effects on Jurkat T cells was determined using the paired T test. Values of *p* < 0.05 were considered significant (*, *p* < 0.05; **, *p* < 0.01; ***, *p* < 0.001; ****, *p* < 0.0001).

## Results

### Gallein, a small molecule inhibitor of Gβγ signaling, enhances TCR-stimulated IL-2 mRNA increases in primary human CD4^+^ T helper cells and Jurkat cells

**Figure 1:**
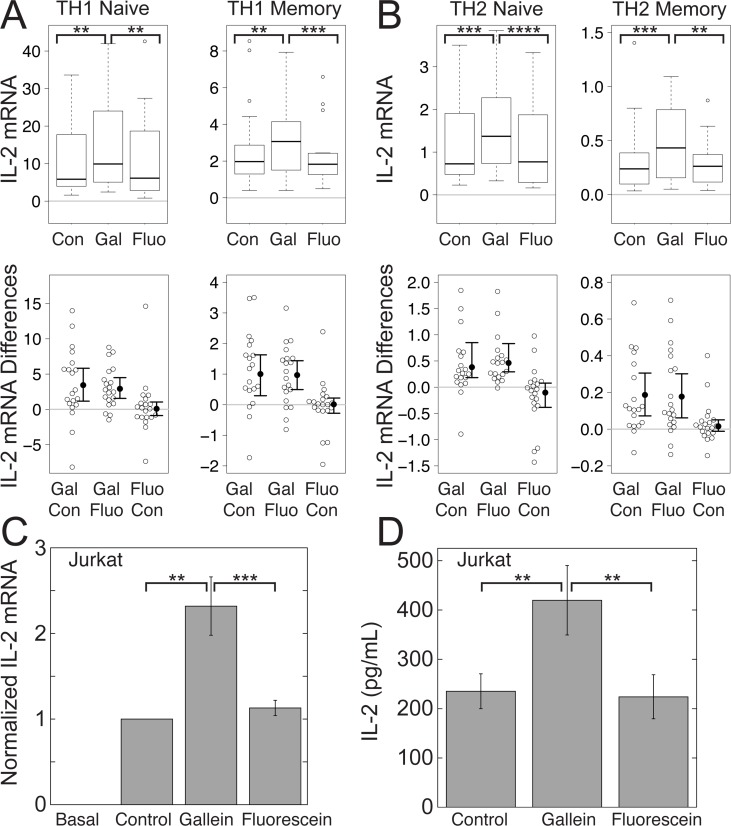
Gβγ inhibition with gallein enhances TCR-stimulated IL-2 increases in CD4^+^ T helper and Jurkat cells. Gallein, but not fluorescein, potentiated TCR-stimulated IL-2 mRNA levels. (A) and (B) Box plots (top) and difference plots (bottom) show data from naïve and memory CD4^+^ T cells isolated from the peripheral blood of 20 healthy donors, stimulated with plate-bound anti-CD3 and soluble anti-CD28, and grown in conditions promoting TH1 (A) or TH2 (B) differentiation in the absence or presence of gallein or fluorescein for three days. IL-2 mRNA levels were determined by qPCR. In the box plots (top), the height of the box plots equals the interquartile range (IQR) and the horizontal line within the box indicates the median value. The whiskers extend to the lowest and highest data points within 1.5 X IQR and the open circles indicate the outliers. In the difference plots (bottom), open circles show pairwise differences in IL-2 mRNA for each sample when treated with the top versus bottom condition listed on the X axis. To the right of the open circles are the median values (closed circles) and 95% confidence intervals. (C) and (D) Jurkat cells were stimulated with plate-bound anti-CD3 and soluble anti-CD28 in the absence or presence of gallein or fluorescein for three days. (C) Data represent the mean ± SE from 17 experiments. IL-2 mRNA was normalized to the amount produced by the TCR-stimulated control. (D) The media was replaced with fresh media containing the same components on the second day, and IL-2 secreted between the second and third days was quantified by ELISA. Data represent the mean ± SE from 6 experiments. **, *p* < 0.01; ***, *p* < 0.001; ****, *p* < 0.0001.

To determine whether Gβγ plays a role in modulating TCR-stimulated IL-2 increases, we tested the effect of gallein, a small molecule inhibitor of Gβγ signaling [22], in primary human CD4^+^ T cells grown for three days in conditions that promote either TH1 or TH2 differentiation and in the Jurkat human CD4^+^ T cell leukemia line, a well-established model system for studying T cell receptor signaling [31]. TH1 cells protect against intracellular organisms, but can also cause inflammation and autoimmune diseases, whereas TH2 cells protect mucosal and epithelial surfaces, but can also cause allergy and asthma [32]. The TCR was stimulated with plate-bound anti-CD3 antibodies and soluble anti-CD28 antibodies for three days. We measured IL-2 mRNA by qPCR, as levels of IL-2 are primarily regulated at the level of transcriptional induction of the IL-2 gene and stability of IL-2 mRNA [33,34]. The levels of IL-2 mRNA were greater in TH1 (Fig. 1A) than in TH2 (Fig. 1B) cells, which is characteristic of these T helper cell subsets [35] and in naïve compared to memory cells (Fig. 1, A and B), which is also consistent with previous observations [36]. Gallein significantly potentiated median TCR-stimulated IL-2 mRNA levels in each of the primary cell lineages tested by 1.6 to 1.9-fold, depending on the T cell subset (Fig. 1, A and B) and mean TCR-stimulated IL-2 mRNA levels in Jurkat cells by 2.4-fold (Fig. 1C). In contrast, fluorescein, a structurally related but inactive compound, had no effect (Fig. 1, A–C). Gallein, but not fluorescein, also increased mean TCR-stimulated levels of IL-2 secreted by Jurkat cells (Fig. 1D). The gallein-dependent increase in secreted IL-2 compared to the control (1.78-fold ± 0.16) was similar to the increase in IL-2 mRNA in cells from the same samples (1.87-fold ± 0.10). M119, a Gβγ inhibitor that is structurally and functionally similar to gallein [22], and which operates by the same mechanism [37], blocks the interactions of Gβγ with downstream effectors, but does not interfere with GPCR-dependent Gα activation or Gα-effector interactions [21]. Our results therefore indicate that Gβγ plays a role in inhibiting TCR-stimulated IL-2 increases that is downstream or independent of GPCR-Gα signaling.

### siRNA-mediated silencing of Gβ_1_ enhances TCR-stimulated IL-2 mRNA levels in primary human CD4^+^ T helper cells and Jurkat cells

**Figure 2:**
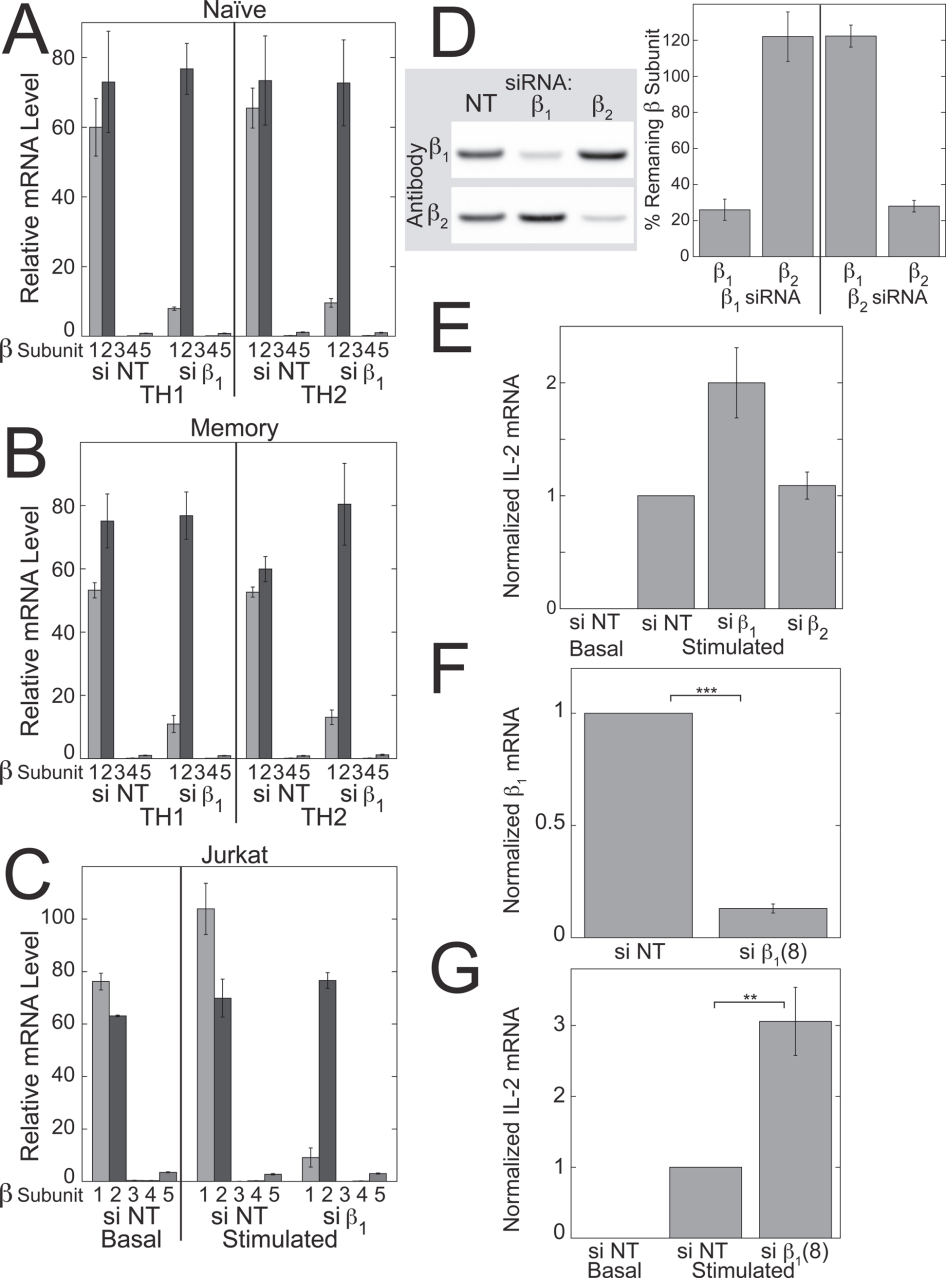
siRNA directed at Gβ_1_ but not Gβ_2_ enhances TCR-stimulated IL-2 mRNA increases in Jurkat cells. Expression of Gβ mRNAs in primary naïve (A) and memory (B) human CD4^+^ T cells grown in TH1 or TH2-promoting conditions, and Jurkat T cells (C) treated with Gβ_1_ siRNA (si β_1_) or NT siRNA (si NT). The primary cells and, where indicated, the Jurkat cells, were stimulated with plate-bound anti-CD3 and soluble anti-CD28 for three days in the presence of the indicated siRNAs. Values represent means ± SE (N = 3). (D) Representative immunoblot (left) and quantification (right) of the effects of Gβ_1_ and Gβ_2_ siRNAs on the protein levels of Gβ_1_ and Gβ_2_ in Jurkat cells. Jurkat cells were treated with the indicated siRNAs for 3 days. Values represent means ± SE (N = 3). (E) Gβ_1_ siRNA but not Gβ_2_ siRNA potentiated TCR-stimulated IL-2 mRNA increases in Jurkat cells. Jurkat cells were stimulated with plate-bound anti-CD3 and soluble anti-CD28 for 3 days in the presence of the indicated siRNAs. Values represent the means ± SE from 17 experiments. The data were normalized to the values for TCR-stimulated cells treated with NT siRNA. (F and G) A second Gβ_1_ siRNA also potentiated TCR-stimulated IL-2 mRNA increases. Values represent the means ± SE from 7 experiments. The data were normalized to the values for TCR-stimulated cells treated with NT siRNA. (F) Gβ_1_ mRNA was measured in stimulated cells. All mRNA levels were determined by qPCR. **, *p* < 0.01; ***, *p* < 0.001.

There are 5 Gβ and 12 Gγ subunit genes in the human genome and results from gene targeting approaches have demonstrated unique physiological roles for certain Gβγ complexes [38]. As the first step in determining whether specific Gβγ complexes are required for modulating TCR-stimulated IL-2 levels in CD4^+^ T helper cells, we identified the Gβ and Gγ subunit mRNAs that are expressed. In primary human CD4^+^ T cells and Jurkat T cells, only a subset of the Gβ and Gγ subunit mRNAs was expressed. Gβ_1_ and Gβ_2_ accounted for >99% of the total Gβ subunit mRNAs in primary naïve (si NT in Fig. 2A) and memory (si NT in Fig. 2B) CD4^+^ T cells, and in Jurkat T cells (si NT in Fig. 2C). Gβ_2_ mRNA was expressed at a slightly higher level than Gβ_1_ mRNA in primary cells, whereas Gβ_1_ mRNA was expressed at a slightly higher level than Gβ_2_ mRNA in Jurkat cells, but these differences were not statistically significant.

In both primary T cells and Jurkat cells, Gβ_1_ siRNA selectively reduced the mRNA level of Gβ_1_ by ∼75–90% (Fig. 2, A–C). At the protein level in Jurkat cells, Gβ_1_ siRNA reduced the level of Gβ_1_, but not Gβ_2_ to 26% of the level in the presence of NT siRNA, and Gβ_2_ siRNA reduced the level of Gβ_2_, but not Gβ_1_ to 26% of the level in the presence of NT siRNA (Fig. 2D).

siRNA directed at Gβ_1_ potentiated TCR-stimulated IL-2 mRNA increases in Jurkat cells by 2-fold (Fig. 2E). A second Gβ_1_ siRNA, si β_1_(8), had similar effects as the first one on Gβ_1_ mRNA levels (Fig. 2F) and TCR-stimulated IL-2 mRNA levels (Fig. 2G). In contrast, siRNA directed at Gβ_2_ did not enhance TCR-stimulated IL-2 mRNA increases (Fig. 2E). These results indicate that Gβ_1_γ complexes play a role in inhibiting TCR-stimulated IL-2 increases.

**Figure 3:**
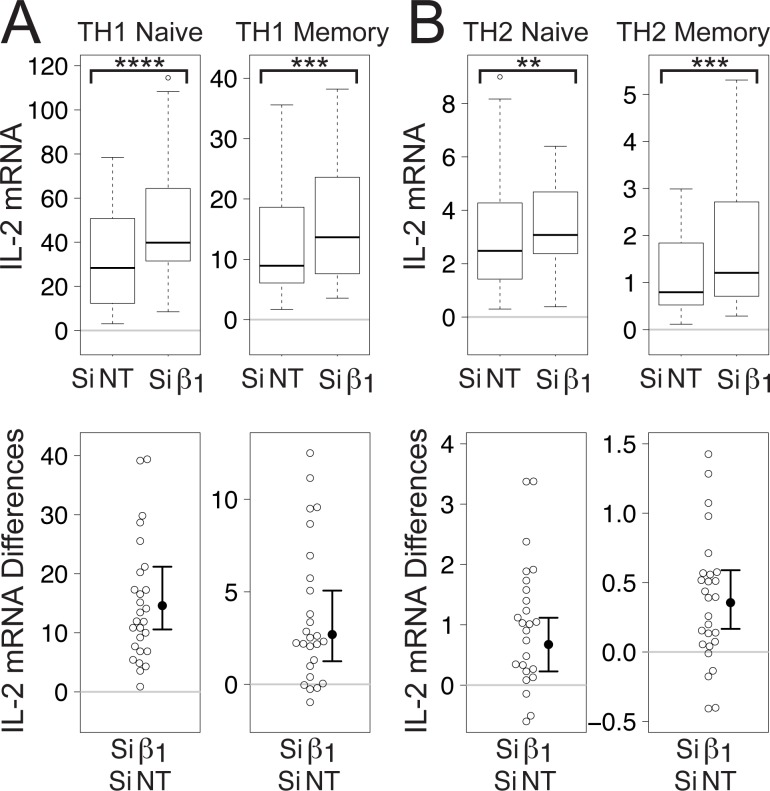
Gβ_1_ siRNA potentiates TCR-stimulated increases in IL-2 mRNA levels in primary human CD4^+^ T cells. Box plots (top) and difference plots (bottom) show data from primary human naïve and memory CD4^+^ T cells isolated from the blood of 30 healthy donors and stimulated for three days with plate-bound anti-CD3 and soluble anti-CD28 in conditions promoting TH1 (A) or TH2 (B) differentiation. IL-2 mRNA levels were determined by qPCR. **, *p* < 0.01; ***, *p* < 0.001; ****, *p* < 0.0001.

As in Jurkat cells, Gβ_1_ siRNA enhanced TCR-stimulated increases in IL-2 mRNA in primary human naïve and memory CD4^+^ T cells grown for three days in conditions promoting differentiation of TH1 (Fig. 3A) and TH2 (Fig. 3B) T helper cell subsets. Again, despite the fact that TCR stimulation caused larger increases in IL-2 mRNA in TH1 cells compared to TH2 cells and in naïve T cells compared to memory T cells, each of the T cell subsets exhibited significant increases in median TCR-stimulated IL-2 mRNA levels (1.2 to 1.5-fold depending on the subset) in response to Gβ_1_ siRNA.

**Figure 4:**
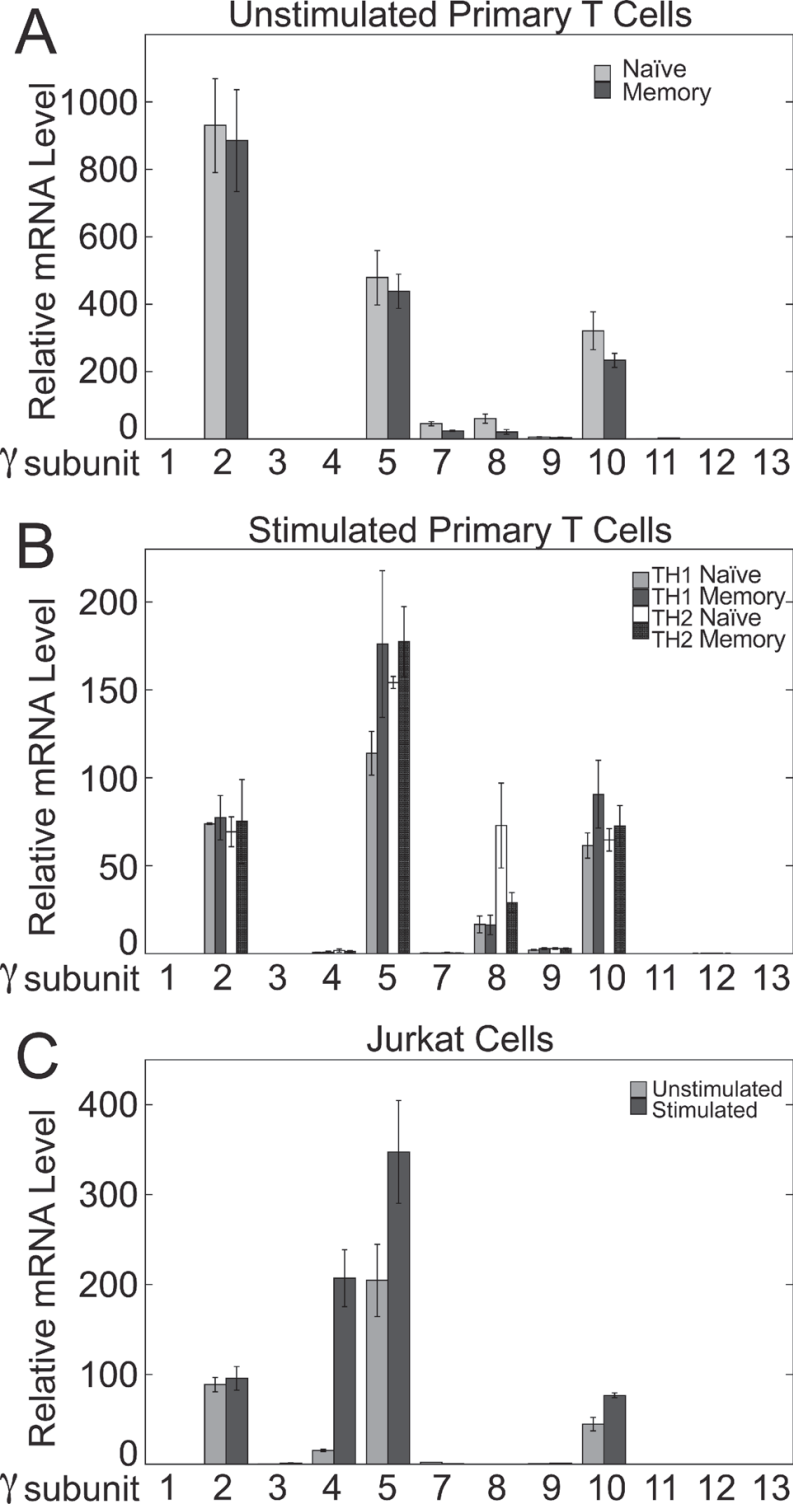
Expression of Gγ subunit mRNAs in primary human CD4^+^ T cells and Jurkat cells. Levels of Gγ mRNAs in unstimulated primary naïve and memory human CD4^+^ T cells (A), stimulated primary naïve and memory human CD4^+^ T cells grown in conditions promoting TH1 or TH2 differentiation (B), and Jurkat cells (C) were determined by qPCR. The stimulated cells were stimulated with plate-bound anti-CD3 and soluble anti-CD28 for 3 days. Values represent the means ± SE from three determinations.

Of the 12 Gγ subunits, the three most predominantly expressed isoforms at the mRNA level in both primary T cells and Jurkat cells were Gγ_2_, Gγ_5_, and Gγ_10_ (Fig. 4). Gγ_8_ was the next most prevalent Gγ subunit in stimulated primary T cells and was expressed at the same level as Gγ_2_ and Gγ_10_ in naïve TH2 cells (Fig. 4B), but was undetectable in Jurkat cells (Fig. 4C). Also, Gγ_4_ was induced robustly in Jurkat cells upon stimulation (Fig. 4C), but was undetectable in unstimulated primary T cells (Fig. 4A) and only minimally detectable in stimulated primary T cells (Fig. 4B). These differences in Gγ subunit mRNA expression between primary T cells and Jurkat cells may indicate differences in the composition of the Gβ_1_γ complexes that inhibit TCR-stimulated IL-2 mRNA increases. However, due to lack of effective antibodies and/or siRNAs, we have not determined the relative importance of particular Gγ subunits for modulating TCR-stimulated IL-2 levels.

### Disrupting Gβγ signaling enhances TCR-stimulated activity of a 300 bp IL-2 promoter

**Figure 5:**
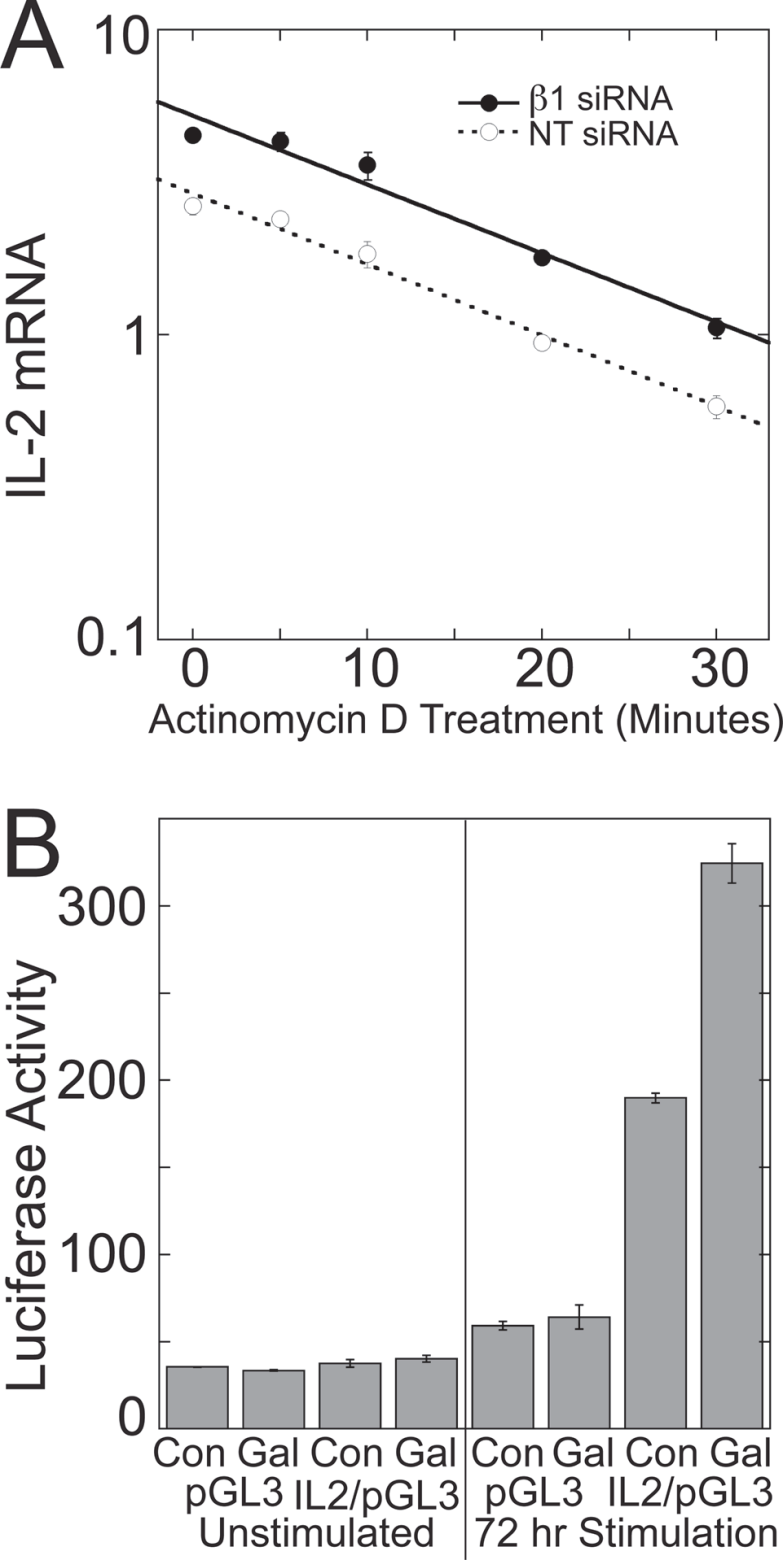
Disrupting Gβγ signaling enhances TCR-stimulated IL-2 transcription without affecting IL-2 mRNA stability. (A) Gβ_1_ siRNA does not increase stability of IL-2 mRNA. After three days of TCR stimulation with plate-bound anti-CD3 and soluble anti-CD28 and treatment with Gβ_1_ siRNA or NT siRNA, Jurkat cells were incubated for the indicated times with 10 μg/mL of Actinomycin D to inhibit transcription, and the rate of IL-2 mRNA degradation was measured. In both cases, the rates of IL-2 mRNA degradation fit a single exponential. Data represent means ± SD from triplicate determinations from a single experiment representative of 5 experiments. (B) Gallein increases IL-2 promoter activity in a luciferase reporter assay. Jurkat cells were stimulated with plate-bound anti-CD3 and soluble anti-CD28 in the presence or absence of gallein for three days following nucleofection with the indicated plasmids. Data represent means ± SD from triplicate determinations from a single assay representative of 8 assays.

Disrupting Gβγ signaling could enhance TCR-stimulated increases in IL-2 mRNA levels by increasing IL-2 transcription and/or IL-2 mRNA stability. To determine whether inhibition of Gβγ signaling increased IL-2 mRNA stability, we measured the half-life of IL-2 mRNA in Jurkat cells stimulated with plate-bound anti-CD3 antibodies and soluble anti-CD28 antibodies for three days and then treated with Actinomycin D to inhibit transcription. Gβ_1_ siRNA did not increase the stability of IL-2 mRNA (Fig. 5A). In the presence of Gβ_1_ siRNA, the t_1/2_ of IL-2 mRNA (11.91 min, SE = 0.58, N = 5) was the same as in the presence of NT siRNA (11.35 min, SE = 0.56, N = 5).

To test whether blocking Gβγ increased IL-2 transcription, the effect of gallein on IL-2 promoter activity was determined using a luciferase reporter plasmid containing a 300 bp region of the IL-2 promoter immediately upstream from the transcription start site, which is sufficient to confer T cell specific inducible transcription of reporter genes [39]. 72 hours of TCR stimulation resulted in a 5-fold increase in luciferase activity in the IL-2 reporter plasmid (IL2/pGL3), but not the empty vector (pGL3) (Fig. 5B). Gallein potentiated this increase by 1.47-fold (*p* < 0.01) (Fig. 5B). Gallein potentiated TCR-stimulated increases in IL-2 mRNA, as quantified by qPCR using portions of the same samples, by 1.56-fold (*p* < 0.001), indicating that increased transcription could account fully for the increased IL-2 mRNA levels.

### Disrupting Gβγ signaling enhances TCR-stimulated NFAT activity

**Figure 6:**
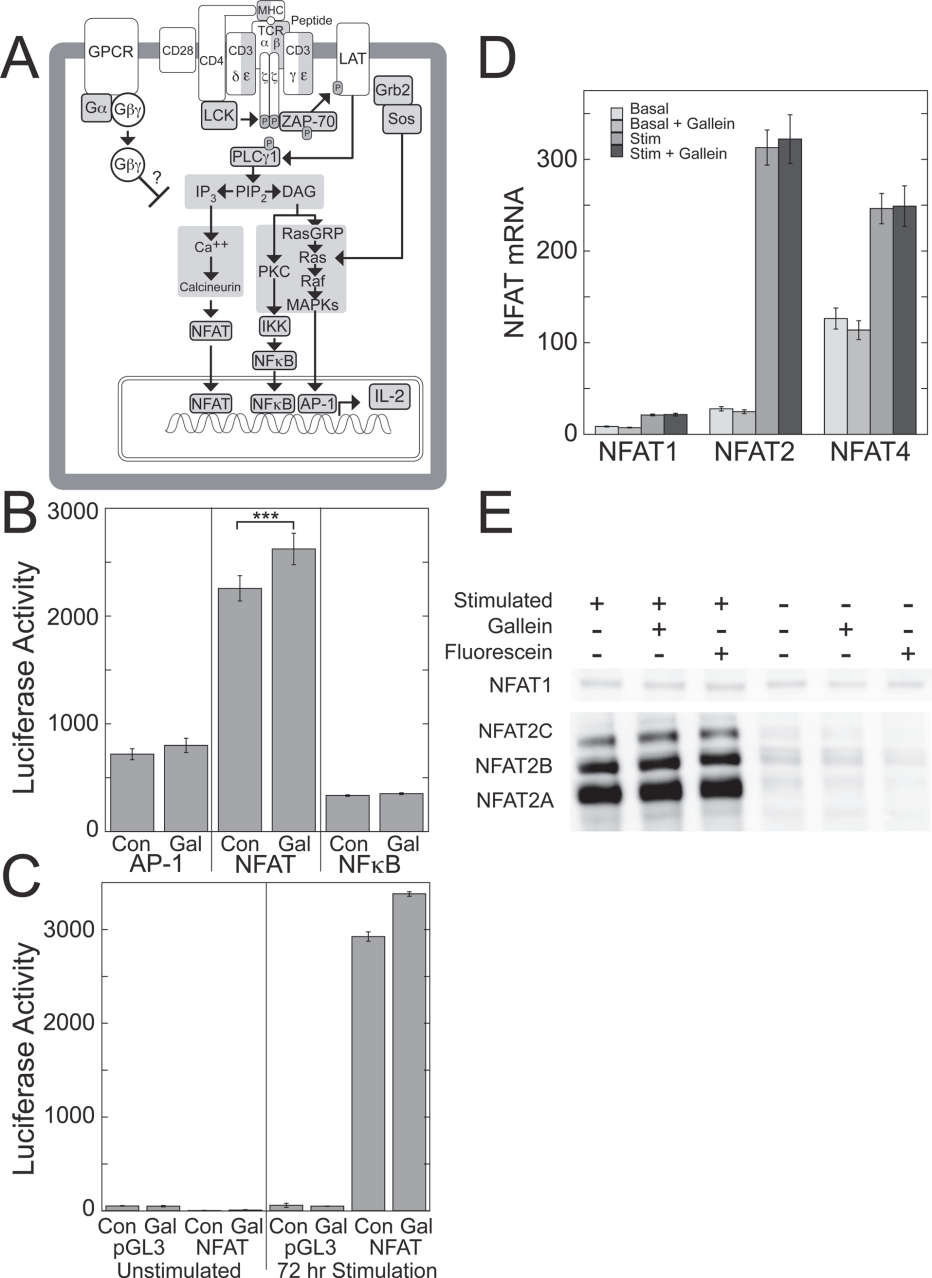
Gallein enhances TCR-stimulated transcriptional activity of NFAT. (A) Major TCR-stimulated pathways leading to IL-2 transcription that could be inhibited by Gβγ. Interactions between the TCR and peptide-major histocompatibility complex (MHC) lead to recruitment of CD4 and its associated kinase, p56-Lck, which phosphorylates tyrosine residues in the cytoplasmic tails of the TCR subunits, leading to recruitment and phosphorylation of the tyrosine kinase, ZAP-70. CD28 costimulation provides an additional signal that is needed for complete T cell activation and regulation of IL-2 production [88]. ZAP-70 and p56-Lck then phosphorylate and activate numerous downstream target proteins, including phospholipase C-γ (PLC-γ), leading to Ras activation, Ca^2+^ increases, cytoskeletal rearrangements, and ultimately, activation of transcription factors that bind to the IL-2 promoter and increase IL-2 transcription. (B-C) Gallein increases transcriptional activity of NFAT, but not AP-1 or NFκB. Jurkat cells expressing reporter plasmids for AP-1, NFAT, or NFκB, were stimulated with plate-bound anti-CD3 and soluble anti-CD28 in the presence or absence of gallein for three days. (B) Data from TCR-stimulated cells expressing the indicated reporter plasmids represent means ± SE from 7 experiments. ***, *p* < 0.001. (C) Data from cells expressing empty vector (pGL3) or the NFAT reporter plasmid represent means ± SD from triplicate determinations in a single experiment representative of 7 experiments. (D) Gallein does not affect mRNA levels of NFAT1, NFAT2, or NFAT4. Portions of the Jurkat cells used for the luciferase assays that measured activity at the NFAT ARRE-2 site were used to measure mRNA levels of NFAT1, NFAT2, and NFAT4 by qPCR. Data represent means ± SE from 7 experiments. (E) Gallein does not cause detectable changes in protein levels of NFAT1 or NFAT2. Jurkat cells were stimulated or not with plate-bound anti-CD3 and soluble anti-CD28 in the absence or presence of gallein or fluorescein. The blot of NFAT1 used lysates from cells stimulated for three days and the blot of NFAT2 used lysates from cells stimulated for two days. Similar results were obtained from cells stimulated for one, two, or three days, and in a second independent experiment.

The 300 bp region of the IL-2 promoter contains binding sites for multiple transcription factors, including activator protein-1 (AP-1), NFAT, and nuclear factor kappa-light chain-enhancer of activated B cells (NF-κB), that play positive roles in IL-2 transcription [40] and could be targets for inhibition by Gβγ (Fig. 6A). To test for regulation of these transcription factors by Gβγ, we determined whether gallein affected their TCR-stimulated activity using luciferase reporter vectors that contained transcription factor binding motifs that monitored activation of the respective transcription factors (Fig. 6B). In Jurkat cells stimulated with plate-bound anti-CD3 antibodies and soluble anti-CD28 antibodies for three days, gallein significantly increased activity of the NFAT reporter by 1.16-fold (Fig. 6, B and C). Gallein did not increase NFAT activity in unstimulated cells (Fig. 6C). The NFAT reporter consisted of the antigen response recognition element-2 (ARRE-2) site in the human IL-2 promoter, which is a composite NFAT/AP-1 site [41]. In contrast, the AP-1 and NF-κB reporters did not exhibit significant changes in TCR-stimulated activity in response to gallein (Fig. 6B). Gallein-dependent increases in TCR-stimulated IL-2 mRNA in portions of the samples used for the NFAT reporter assay (1.73-fold, *p* < 0.01) were larger than the increases in NFAT activity. Therefore, while these results suggest that increased NFAT activity resulting from Gβγ inhibition contributes to increased IL-2 transcription, this increased NFAT activity is unlikely to account entirely for the increased IL-2 transcription. Additional changes in response to Gβγ inhibition are likely to be involved as well.

### Blocking Gβγ does not affect NFAT expression

The three NFAT family members that are expressed in T cells are NFAT1, NFAT2, and NFAT4 [42]. NFAT2 and NFAT4 were expressed at higher mRNA levels than NFAT1, and NFAT2 mRNA exhibited the greatest fold increase upon TCR stimulation in Jurkat cells (Fig. 6D). Gallein did not affect the mRNA levels of any of these NFAT family members before or after three days of TCR stimulation (Fig. 6D).

To investigate how inhibition of Gβγ signaling potentiates TCR-stimulated NFAT activity, we focused on NFAT1 and NFAT2 for two reasons. First, NFAT4 is expressed primarily in immature thymocytes and nonlymphoid tissues rather than in peripheral T cells [42]. Second, although overexpressed NFAT1, NFAT2, and NFAT4 can all activate transcription at the ARRE-2 site in the IL-2 promoter, NFAT4 binds to this site with lower affinity than NFAT1 and NFAT2 and does not appear to be part of the endogenous NFAT complex on this site, suggesting that the site is not a physiological DNA binding site for NFAT4 [43].

Gallein did not cause detectable changes in the protein expression levels of NFAT1 or NFAT2 before or after 1–3 days of TCR stimulation (Fig. 6E). NFAT1 exhibited minimal induction upon TCR stimulation, as observed previously [44], and gallein did not affect its protein expression level (Fig. 6E). Immunoblots of NFAT2 revealed three bands, which appear to correspond to isoforms A, B, and C, and which exhibited large increases in expression upon TCR stimulation, as previously demonstrated [44,45]. Gallein did not affect the expression levels of any of these isoforms (Fig. 6E).

### Disrupting Gβγ signaling enhances TCR-stimulated nuclear localization of NFAT1

Because nuclear translocation of NFAT1 and NFAT2 in response to dephosphorylation by Ca^2+^/calmodulin-activated calcineurin is a major mechanism for NFAT activation [46], we investigated whether the effects of gallein on TCR-stimulated IL-2 transcription and NFAT activity were the result of increases in the nuclear localization of NFAT1 and/or NFAT2. To this end, we tested whether gallein increased the nuclear localization of fusions of GFP to NFAT1 and NFAT2 in Jurkat cells stimulated with plate-bound anti-CD3 and soluble anti-CD28 for three days.

**Figure 7:**
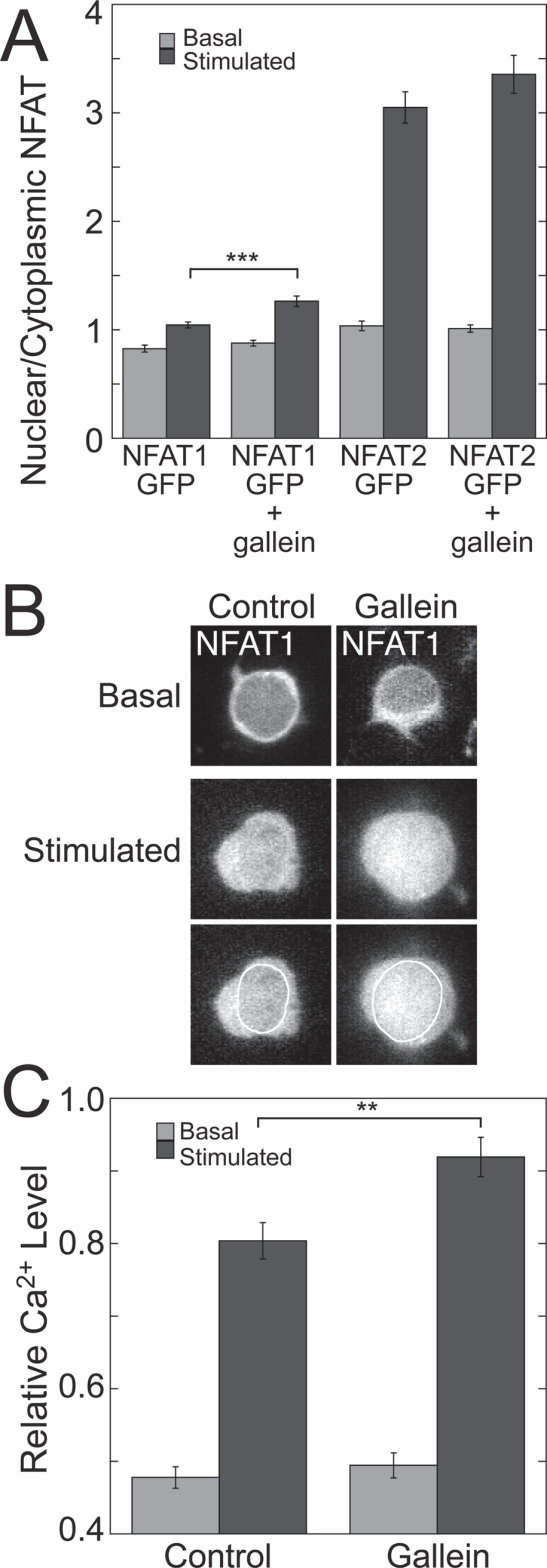
Gallein potentiates TCR-stimulated increases in nuclear localization of NFAT1 and intracellular Ca^2+^. (A) Quantitation of the ratio of nuclear to cytoplasmic NFAT1-GFP and GFP-NFAT2 in basal and stimulated Jurkat cells in the presence or absence of gallein. Cells were stimulated with plate-bound anti-CD3 and soluble anti-CD28 for three days. Data represent means ± SE from 130–162 cells for each condition. ***, *p* < 0.001. (B) Representative images of NFAT1-GFP in basal and stimulated cells in the presence or absence of gallein. In the lower of the two rows of stimulated cells the nuclear borders are outlined in white. (C) Gallein potentiates TCR-stimulated increases in intracellular Ca^2+^ after three days of TCR stimulation. Relative Ca^2+^ levels were determined using R-GECO-mCerulean as described in Materials and Methods. Data represent the means ± SE from > 320 cells for each stimulated condition and > 200 cells for each unstimulated condition. **, *p* < 0.01.

TCR stimulation significantly increased nuclear localization of both NFAT1-GFP and GFP-NFAT2, with a greater effect on GFP-NFAT2 (Fig. 7A), consistent with the previous observation that submaximal Ca^2+^ increases cause preferential nuclear localization of NFAT2 compared to NFAT1 [47]. Gallein significantly enhanced TCR-stimulated nuclear localization of NFAT1-GFP by 1.21-fold (Fig. 7A). In contrast, TCR-stimulated nuclear localization of GFP-NFAT2 exhibited a smaller increase in response to gallein that was not significant (Fig. 7A). Gallein did not affect localization of NFAT1-GFP or GFP-NFAT2 significantly in the absence of TCR stimulation. Fig. 7A shows quantitation of NFAT1-GFP and GFP-NFAT2 localization in basal and stimulated cells in the presence or absence of gallein. Fig. 7B shows representative images of NFAT1-GFP in basal and stimulated cells in the presence or absence of gallein.

### Disrupting Gβγ signaling enhances TCR-stimulated increases in intracellular Ca^2+^

As NFAT1 and NFAT2 translocate to the nucleus as a result of Ca^2+^/calmodulin-dependent activation of calcineurin, we investigated whether inhibiting Gβγ with gallein enhanced TCR-stimulated increases in intracellular Ca^2+^. We used a calcium indicator, mCerulean-R-GECO1, consisting of a fusion of the red fluorescent Ca^2+^ sensor, R-GECO1 [27], to the fluorescent protein, mCerulean [28], so that R-GECO1 fluorescence could be normalized to the expression level of the plasmid. TCR stimulation for three days resulted in a 1.7-fold increase in intracellular Ca^2+^ (Fig. 7C). Inhibiting Gβγ with gallein significantly potentiated intracellular Ca^2+^ in TCR-stimulated cells by a factor of 1.13-fold (Fig. 7C), which may contribute to the effects on NFAT transcriptional activity (Fig. 6, B and C) and nuclear localization of NFAT1-GFP (Fig. 7, A and B). The modest size of this gallein-induced Ca^2+^ increase did not result from saturation of mCerulean-R-GECO1, because in response to stimulation of Jurkat cells with 5 μM ionomycin and 2 mM CaCl_2_, the relative Ca^2+^ level detected by the sensor was 1.86-fold higher than that in TCR-stimulated cells treated with gallein. Gallein did not increase intracellular Ca^2+^ in the absence of TCR stimulation (Fig. 7C).

### The potentiating effect of Gβγ inhibition on IL-2 transcription requires continuous Gβγ inhibition for at least two days of TCR stimulation

**Figure 8:**
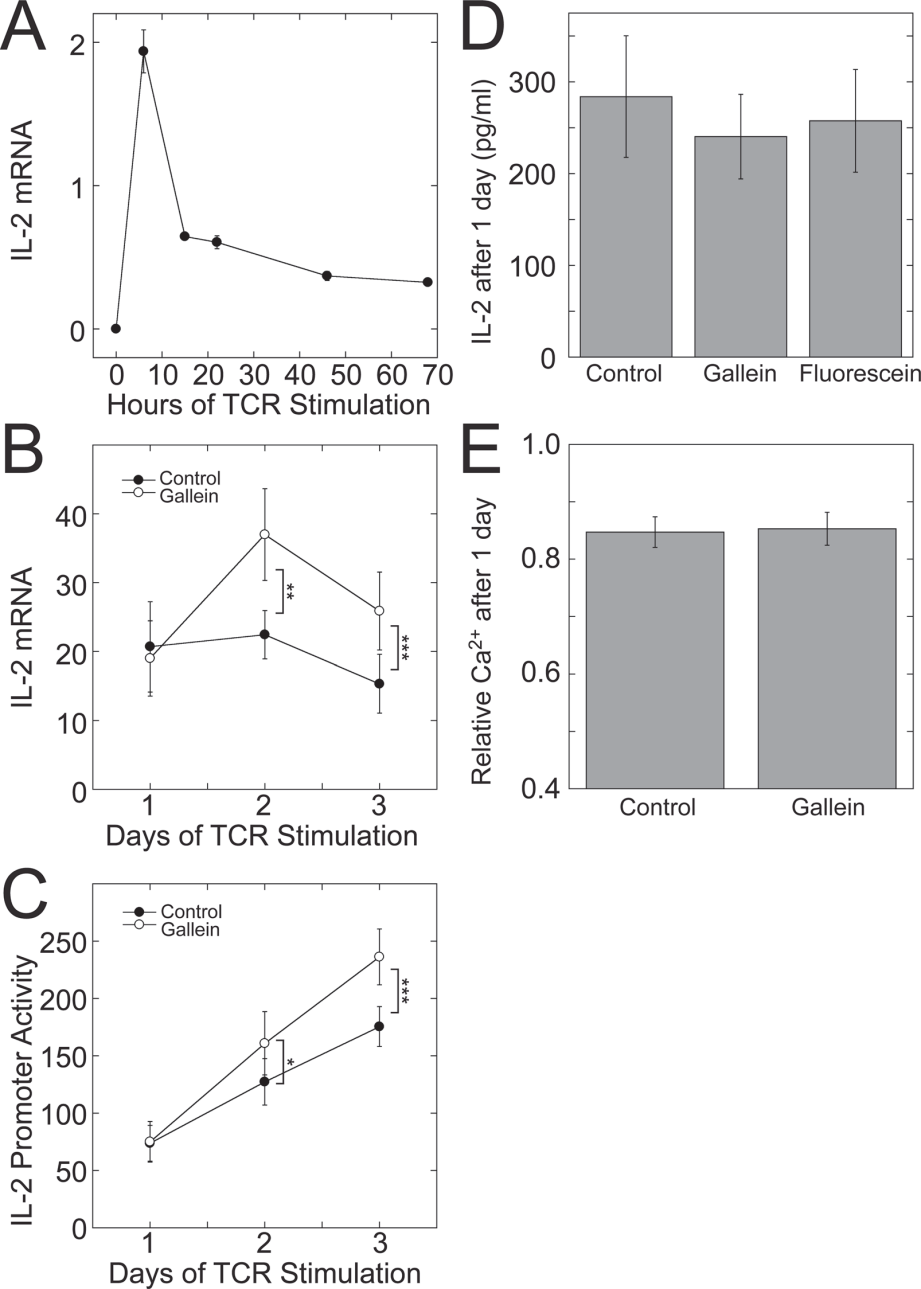
Potentiation of IL-2 mRNA and Ca^2+^ increases by gallein requires two days of TCR stimulation. (A) IL-2 levels peaked within 24 hours of TCR stimulation and then decreased over the next 48 hours. Jurkat cells were stimulated with plate-bound anti-CD3 and soluble anti-CD28 antibodies and IL-2 mRNA levels were determined by qPCR at the indicated times. Data represent the means ± SD from a single experiment that is representative of three such experiments. (B) TCR-stimulated IL-2 mRNA increases and (C) activity at the minimal 300-bp IL-2 promoter were not potentiated by gallein until after 2–3 days of TCR stimulation. IL-2 promoter activity in (C) was determined in luciferase assays using the same cells in which IL-2 mRNA was measured in (B). Jurkat cells were stimulated with plate-bound anti-CD3 and soluble anti-CD28 antibodies in the presence or absence of gallein for the indicated times. Data points represent the means ± SE of 8 experiments. *, *p* < 0.05; **, *p* < 0.01; ***, *p* < 0.001. (D) IL-2 secretion was not increased by gallein after one day of TCR stimulation. Jurkat cells were stimulated with plate-bound anti-CD3 and soluble anti-CD28 antibodies for one day in the absence or presence of gallein or fluorescein, and IL-2 secreted into the media was quantified by ELISA. Data points represent the means ± SE of 6 experiments. (E) Intracellular Ca^2+^ was not increased by gallein after one day of TCR stimulation. Relative Ca^2+^ levels were determined using R-GECO-mCerulean as described in Materials and Methods. Data represent the means ± SE from > 330 cells for each condition.

Ligation of the TCR and CD28 prompts CD4^+^ cells to secrete IL-2 rapidly, which further enhances their proliferation and survival [48]. However, the levels of IL-2 decrease as the cells start to differentiate [34,39]. Accordingly, we observed an initial peak of IL-2 mRNA within 24 hours of TCR stimulation of Jurkat cells with plate-bound anti-CD3 antibodies and soluble anti-CD28 antibodies that decreased upon further stimulation (Fig. 8A). To determine whether the potentiating effect of Gβγ inhibition on TCR-stimulated IL-2 transcription required three days of TCR stimulation, we measured IL-2 mRNA and activity at the 300 bp IL-2 promoter each day over a three-day period of TCR stimulation. Gallein only potentiated TCR-stimulated IL-2 mRNA levels (Fig. 8B) and IL-2 promoter activity (Fig. 8C) after 2–3 days of TCR stimulation. Consistent with these results, IL-2 secreted during the first day of TCR stimulation was not increased by gallein (Fig. 8D), in contrast to IL-2 secreted between the second and third days of TCR stimulation (Fig. 1D). Additionally, intracellular Ca^2+^ in cells that had been stimulated at the TCR for one day was not increased by gallein (Fig. 8E), in contrast to cells that had been stimulated at the TCR for three days (Fig. 7C). Although by 24 hours IL-2 mRNA levels had decreased from an initial peak (Fig. 8A), activity at the IL-2 promoter increased linearly over the three days (Fig. 8C), in agreement with previous observations that the decrease in IL-2 mRNA levels after the peak reflects decreased mRNA stability [34].

**Figure 9:**
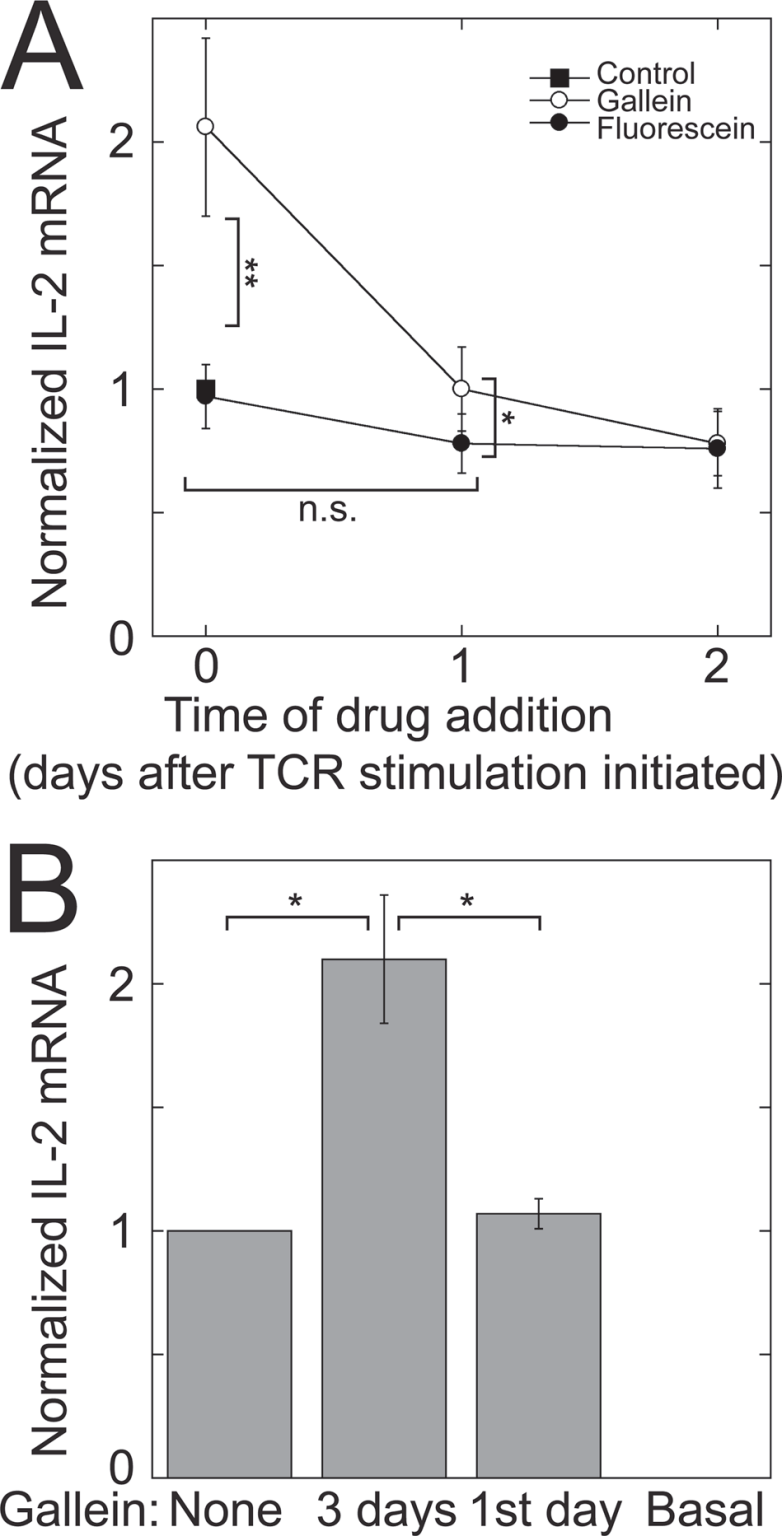
Potentiation of IL-2 mRNA increases requires continuous Gβγ inhibition during prolonged TCR stimulation. (A) Gallein had minimal or no effect on IL-2 mRNA levels when added after the first day of TCR stimulation. Jurkat cells were stimulated with plate-bound anti-CD3 and soluble anti-CD28 antibodies for three days. Gallein or fluorescein was added from the beginning of TCR stimulation (0 days) or at the indicated times afterwards. IL-2 mRNA levels were determined by qPCR after three days of TCR stimulation. Data represent means ± SE from 5 experiments and were normalized to the value of the untreated control. n.s., not significant; *, *p* < 0.05; **, *p* < 0.01. (B) Treatment with gallein for only the first day of a three-day TCR stimulation was not sufficient to potentiate TCR-stimulated IL-2 mRNA levels. Jurkat cells were stimulated with plate-bound anti-CD3 and soluble anti-CD28 antibodies for three days in the absence of gallein (none), or in the presence of gallein for the full three days or only the first day. In all cases, the media was changed after the first day and replaced with media that contained anti-CD28 and either gallein (three days), or no gallein (none and first day). Data represent the means ± SE from 4 experiments and were normalized to the untreated control. *, *p* < 0.05.

The requirement of prolonged TCR stimulation for a potentiating effect of Gβγ inhibition on IL-2 transcription could indicate that Gβγ must be blocked continuously for the duration of TCR stimulation. Alternatively, there could be a specific interval during TCR stimulation in which blocking Gβγ has an effect. To distinguish between these possibilities, we stimulated the TCR in Jurkat cells for three days and added gallein at various times, either from the beginning of TCR stimulation or after one or two days of stimulation (Fig. 9A). If gallein was added after the first day of TCR stimulation, it had minimal or no effect on IL-2 mRNA levels. This result indicates that blocking Gβγ during the first day of TCR stimulation causes change(s) that are required for the increased levels of IL-2 mRNA that are observed after 2–3 days of TCR stimulation. However, treatment with gallein for just the first day was not sufficient. In cells that were stimulated at the TCR for three days, but treated with gallein for only the first day, after which it was removed, IL-2 mRNA levels were the same as in cells that had not been treated with gallein at all (Fig. 9B). These results suggest that continuous inhibition of Gβγ for at least two days of TCR stimulation is required for a potentiating effect on IL-2 transcription.

## Discussion

GPCR-G protein signaling modulates a vast array of cellular processes and these signaling pathways are targets for pharmaceuticals that treat cardiovascular, central nervous system, endocrine, and urogenital disorders [49], but, less frequently, immunological disorders [2]. Although Gα subunits have traditionally been thought to be responsible for much of the signaling downstream from GPCRs, the list of functions regulated by Gβγ complexes continues to grow [19,20]. The results presented here indicate that Gβγ complexes play a role in inhibiting TCR-stimulated IL-2 transcription, suggesting that they could be useful drug targets for treating autoimmune disorders in which modest increases in IL-2 have been shown to be beneficial, such as chronic graft-versus-host disease [8] and hepatitis C virus-induced vasculitis [9].

Blocking Gβγ signaling, either with the small molecule Gβγ inhibitor, gallein, or with Gβ_1_ siRNA led to potentiation of TCR-stimulated IL-2 transcription, indicating that Gβ_1_γ plays an inhibitory role. Further experiments with gallein demonstrated that Gβγ inhibits TCR-stimulated Ca^2+^ increases, nuclear localization of NFAT1, and NFAT activity. These are direct effects of Gβ_1_γ complexes rather than Gα subunits, because gallein exclusively blocks Gβγ complex interactions with effectors. Gallein belongs to a class of Gβγ inhibitors that operate using the same reversible noncovalent mechanism [37], of which M119, the first identified member, has been the most extensively studied. The specificity of M119 for blocking Gβγ and not Gα was demonstrated by inhibition of fMLP receptor-stimulated Ca^2+^ increases mediated by Gβγ from G_i_, but not M3-muscarinic receptor-stimulated Ca^2+^ increases mediated by Gα_q_ from G_q_ [21]. Moreover, M119 does not promote dissociation of Gα_i_ from Gβγ [21].

Our observation that potentiation of IL-2 transcription required continuous Gβγ inhibition during at least two days of TCR stimulation and was obtained only after IL-2 levels had decreased from an initial peak may indicate that Gβγ signaling plays a role in the negative feedback mechanisms that result in the transient nature of IL-2 secretion in TCR-stimulated CD4^+^ T cells [50–53]. Moreover, the delayed effect of gallein, which contrasts with previously described effects that generally occurred in a matter of minutes to hours [22], may indicate that the effect of Gβγ inhibition on TCR-stimulated IL-2 transcription involves induction or repression of signaling proteins during T cell activation and differentiation.

The potentiating effect of Gβγ inhibition on TCR-stimulated nuclear localization of NFAT1 may result from enhancement of TCR-mediated increases in intracellular Ca^2+.^ There is precedent for this effect of Gβγ inhibition in that pretreatment of activated primary T lymphocytes with gallein resulted in increased levels of intracellular Ca^2+^ upon stimulation with CXCL11 [54]. The mechanism by which Gβγ inhibition can enhance increases in intracellular Ca^2+^ in T cells remains to be determined, but may involve L-type voltage-dependent Ca^2+^ (Ca_V_1) channels. Gβγ can block activation of Ca_V_1 channels [55–57], which are expressed in primary human T cells and Jurkat cells, and which are important for Ca^2+^-mediated NFAT translocation to the nucleus and IL-2 production [58,59]. Moreover, gallein has been demonstrated to prevent inhibition of Ca_V_1 channels by Gβγ [57]. Ca_V_1 channels in T cells are activated by the TCR by an unknown mechanism, rather than by T cell depolarization [59]. This requirement of TCR stimulation for Ca_V_1 channel activation is consistent with our observation that Gβγ inhibition enhances TCR-stimulated IL-2 transcription but has no effect in the absence of TCR stimulation.

The greater magnitude of the effect of Gβγ inhibition on TCR-stimulated IL-2 transcription compared to that on TCR-stimulated Ca^2+^ increases, and nuclear localization and transcriptional activity of NFAT suggests that modulation of additional pathway(s) that regulate IL-2 transcription is involved. As gallein/M119 does not prevent interaction of Gβγ with N-type Ca^2+^ channels, inwardly rectifying K^+^ (GIRK) channels, ERK1/2, or the adenylyl cyclase isoforms ACII, IV, and V1 [60], these effectors cannot account for the ability of gallein to enhance TCR-stimulated IL-2 transcription. In contrast, Gβγ interaction with and activation of PLCβ2/PLCβ3, pREX guanine nucleotide exchange factor (specific for Rac), PI3Kγ, and G protein-coupled receptor kinase 2 (GRK2) can be inhibited by gallein/M119 [60]. However, current evidence does not support a role for these effectors in mediating Gβγ-dependent inhibition of TCR-stimulated IL-2 increases.

The above Gβγ-regulated effectors appear unlikely to mediate Gβγ-dependent inhibition of TCR-stimulated IL-2 increases for the following reasons. Although PLC-γ plays an important role in T cell activation downstream of the TCR [61], PLC-β2 and PLC-β3 are important for chemotaxis of lymphocytes but not for TCR-mediated T cell activation [62,63]. Moreover, when the chemokine stromal cell derived factor-1α (SDF-1α) stimulates association of its receptor, CXCR4, with the TCR, TCR-stimulated IL-2 production is enhanced rather than inhibited [64]. Regarding P-Rex1, inhibition of Gβγ-mediated stimulation of this exchange factor is unlikely to account for potentiation of TCR-stimulated IL-2 transcription by gallein as thymocytes from mice with Rac1/Rac2 double knockouts exhibit decreased rather than increased TCR-stimulated IL-2 production [65]. Similarly, the following results make it unlikely that inhibiting activation of PI3Kγ by Gβγ would lead to enhancement of TCR-stimulated IL-2 increases. A PI3Kγ selective inhibitor did not affect IL-2 production in response to anti-CD3 and anti-CD28 stimulation of naïve T cells from murine lymph nodes [66], T cells from PI3Kγ knockout mice exhibited decreased [67] or no change [68] in IL-2 production in response to anti-CD3 and anti-CD28 stimulation, and CD4^+^ T cells from PI3Kγ kinase-dead knock-in mice produced decreased levels of production of IL-2 in response to anti-CD3 and anti-CD28 stimulation [69]. Finally, there is no simple scenario involving GRK2/3-GPCR regulation that can account for the similar effects of gallein and Gβ_1_ siRNA. Upon GPCR-G protein activation, Gβγ can bind to the PH domains of GRK2/GRK3, causing translocation to the plasma membrane, and GPCR phosphorylation and desensitization [70]. Blocking interaction between Gβγ and GRK2/3 with gallein might increase signaling of a GPCR that can enhance TCR-stimulated IL-2 transcription, but Gβ_1_ siRNA would be predicted to decrease rather than increase signaling of this GPCR. Further studies will be needed to identify the effector protein(s) that mediate Gβγ-dependent inhibition of TCR-stimulated IL-2 increases.

There is precedent for transcriptional regulation by Gβγ complexes, both stimulatory and inhibitory. These effects generally involved Gβγ localized to the nucleus [71–75]. As an example of transcriptional inhibition by Gβγ, Gβ_1_γ_2_ co-localized with AP-1 complexes in the nuclei of HEK-293 cells and inhibited AP-1 activity by recruitment of histone deacetylases (HDACs) [74]. Additionally, Gβ_1_ and Gβ_2_ interacted with the glucocorticoid receptor and suppressed its transcriptional activity in the nuclei of HCT116 cells [73]. In contrast, as an example of transcription activation by Gβγ, Gβ_2_γ_12_ translocated into the nuclei of HEK293 cells upon stimulation of the angiotensin II type 1 receptor and associated with the transcription factor MEF2A, histones H2B and H4, and HDAC5, and depletion of Gβ_2_ decreased the activity of MEF2A [75]. In addition, Gβ_1_γ_2_ bound to HDAC5 in total cell lysates of rat heart and HEK293A cells and inhibited its transcriptional co-repression activity, leading to activation of MEF2C [76]. Although we observed GPCR-dependent internalization of Gβγ from the plasma membrane to vesicles in the cytoplasm in HEK-293 cells that exhibited partial overlap with Rab11-labeled endosomes [77], indicating potential roles for Gβγ internal to the plasma membrane, the vast majority localized outside of the nucleus, although we cannot rule out the presence of minor amounts there. Subsequently, GPCR-dependent translocation of Gβγ to other endomembranes including the Golgi complex and the endoplasmic reticulum, but not to the nucleus, was also reported [78].

One or more GPCRs could stimulate release of the Gβγ that inhibits TCR-stimulated IL-2 transcription. The ligands for these GPCRs would have to be produced by the T cells themselves or be present in the serum, because the cells were cultured in the absence of other cells that could provide ligands in vivo, such as dendritic cells. Of the GPCRs known to inhibit production of IL-2, the A2A-adenosine receptor [12], the μ opioid receptor [14], and the CB1 and CB2 cannabinoid receptors [15] appear to achieve this exclusively by increasing cAMP levels. As gallein does not prevent Gβγ from interacting with adenylyl cyclase isoforms ACII, ACIV, and ACV1 [60], these receptors are unlikely to be involved, but the Edg-4/LPA2 receptor [17] and the β_2_-adrenergic receptor [13] remain as potential candidates. Alternatively, the TCR or a tyrosine kinase receptor could transactivate a GPCR [79]. Finally, the direct involvement of Gβγ in regulating cytokine expression does not necessarily implicate a GPCR [80]. For instance, in the absence of GPCR stimulation, AGS family proteins can activate Gβγ [81].

The potentiation of TCR-stimulated IL-2 production that results from Gβγ inhibition suggests that Gβγ could be a useful drug target for treating autoimmune diseases, as low dose IL-2 therapy has been shown to effectively suppress immune responses in chronic graft-versus-host disease [8] and hepatitis C virus-induced vasculitis [9]. Further experiments using in vivo models of autoimmune diseases would be required to test this hypothesis. Given the involvement of Gβγ signaling in so many essential physiological processes [20], it might seem that blocking Gβγ systemically would be inadvisable. However, there is precedent for therapeutic efficacy without untoward side effects in animal models of acute inflammation [22], pain [21], and heart failure [23]. Additionally, adoptive transfer of genetically modified T cells, a promising approach for treating cancer and persistent viral infections [82], could potentially be used to provide T cells with disrupted Gβγ signaling.

Blocking Gβγ in T cells could have the additional benefit for autoimmune diseases of preventing localization of autoreactive T cells at inflammatory sites. For instance, gallein blocks CXCL11 induced migration of activated T lymphocytes [54]. CXCR3, for which CXCL11 is a ligand, is likely to mediate the infiltration of T cells into the synovial tissue of rheumatoid arthritis patients, because this receptor is highly expressed in CD4^+^ T cells that accumulate in their synovial tissue [83], and accumulation of these T cells in the synovium is associated with expression of CXCR3 ligands by synovial fibroblasts [84,85]. As pharmacological blockade of lymphocyte traffic is effective for treating multiple sclerosis and Crohn’s disease [86,87], inhibiting Gβγ might have applications for these diseases as well.
